# Cat and Dog Exposures to Cocaine or Methamphetamine

**DOI:** 10.1001/jamanetworkopen.2024.51833

**Published:** 2024-12-20

**Authors:** Orrin D. Ware, Renee Schmid

**Affiliations:** 1 School of Social Work, University of North Carolina at Chapel Hill; 2 Pet Poison Helpline/SafetyCall International LLC, Bloomington, Minnesota

## Abstract

This cross-sectional study examines trends in cat and dog exposures to cocaine or methamphetamine using data from a specialty animal poison control center in the US.

## Introduction

Cocaine and methamphetamine are the most common illicit stimulants in the US, with 1.8% and 0.9% of individuals, respectively, using them in the past 12 months.^1^ In a study examining confirmed cases of drugs of abuse, cocaine and methamphetamine were in the top 5 substance exposures among cats and dogs.^2^ Considering the involvement of cocaine and methamphetamine in overdose deaths among humans in the US,^3^ this study examined trends in cat and dog exposures to these substances using data from a specialty animal poison control center. This study is critical because it raises awareness about the potential consequences of unsecured illicit stimulants. The importance of veterinary medicine cannot be understated, as cats and dogs are crucial members of households,^4^ often providing comfort and emotional support, highlighting the intrinsic relationship between human and animal health.

## Methods

The University of North Carolina at Chapel Hill institutional review board indicated that this cross-sectional study did not require ethical review because human participants were not involved. Additionally, the University’s Office of Animal Care and Use also stated ethical review was not required because neither live animals nor animal tissues were acquired for this study, and the data were already gathered from the organization for purposes that were unrelated to the current work. This study followed the STROBE reporting guideline.

This study used deidentified secondary data from calls about potential cat and/or dog exposures to cocaine or methamphetamine from 2019 to 2023 to Pet Poison Helpline, an international specialty animal poison control center. Pet owners, family members, veterinary facilities, and law enforcement personnel may initiate calls concerning a potential exposure. Data for this study included the animal’s age in months, the animal’s weight, the geographic region of the call (Canada and the US), and annual count data. Trend analyses were conducted using the National Cancer Institute Joinpoint Regression Program 5.0.2 in July 2024. Joinpoint regression was used to examine annual count data with specifications including constant variance, the maximum number of Joinpoints set to 0, and the model selected by bayesian information criterion. Trend analyses were conducted for 6 groups: (1) cats exposed to cocaine, (2) cats exposed to methamphetamine, (3) cats exposed to cocaine or methamphetamine, (4) dogs exposed to cocaine, (5) dogs exposed to methamphetamine, and (6) dogs exposed to cocaine or methamphetamine. Two-sided *P* < .05 was considered statistically significant.

## Results

The Table lists the study sample’s characteristics, including 63 cats and 433 dogs. Approximately 65% (n = 41) of the cat sample was exposed to cocaine, and 58% (n = 250) of the dog sample was exposed to methamphetamine. The Figure shows the output from the Joinpoint regression. Three trends were statistically significant: (1) cats exposed to cocaine (Annual Percent Change [APC], 51.93 [95% CI, 1.04-128.46]; *P* = .047), (2) dogs exposed to cocaine (APC, 39.00 [95% CI, 7.07-80.45]; *P* = .03), and (3) dogs exposed to cocaine or methamphetamine (APC, 37.68 [95% CI, 15.28-64.43]; *P* = .01). The significant APC for dogs exposed to cocaine or methamphetamine appears driven by the dogs exposed to cocaine and not those exposed to methamphetamine.

**Table.** Characteristics of the Study Sample

Characteristics	Cocaine or Methamphetamine exposure, No. (%)	
	Cats (n = 63)	Dogs (n = 433)
Age, mean (SD), mo	41.1 (51.6)	39.9 (41.4)
Weight, mean, SD, kg	4.6 (1.5)	15.0 (12.4)
Exposure		
Cocaine	41 (65.1)	183 (42.3)
Methamphetamine	22 (34.9)	250 (57.7)
Geographic region		
Canada: Atlantic Region	0 (0.0)	1 (0.2)
Canada: Central Canada	6 (9.5)	30 (6.9)
Canada: Prairie Provinces	3 (4.8)	8 (1.8)
Canada: West Coast	3 (4.8)	4 (0.9)
United States: Midwest	6 (9.5)	63 (14.5)
United States: Northeast	10 (15.9)	49 (11.3)
United States: South	18 (28.6)	115 (26.6)
United States: West	17 (27.0)	163 (37.6)

**Figure.**
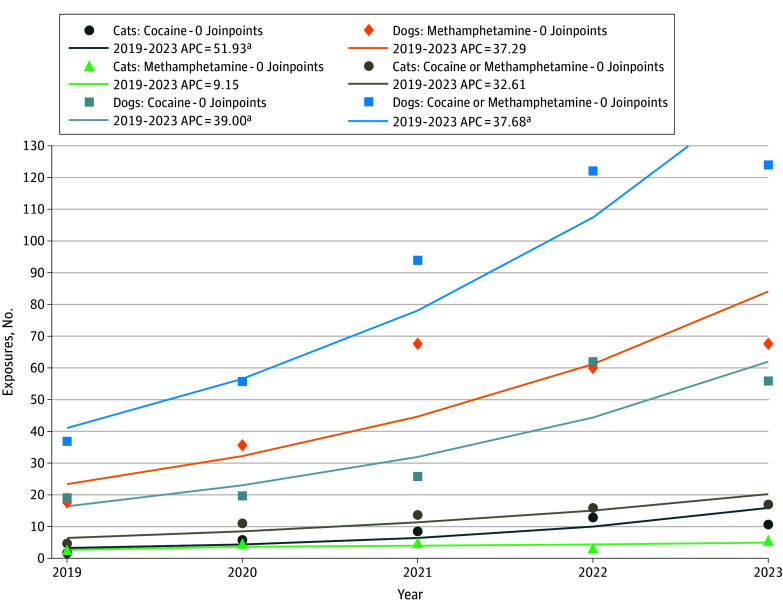
Cat and Dog Exposures to Cocaine and Methamphetamine: 2019 to 2023 Joinpoint regression analysis of cat and dog exposures to cocaine and methamphetamine from 2019 to 2023 using data from a specialty animal poison control center. ^a^*P* < .05.

## Discussion

This study found no significant trends of methamphetamine exposure for either cats or dogs reported using data from an international animal poison control center. However, significant increases in cocaine exposure were identified. Animals are highly sensitive to the stimulatory and sympathomimetic effects of cocaine and methamphetamine. Any degree of exposure should be considered concerning, as there is the risk of severe toxicity and death,^5,6^ even with aggressive medical therapy. It is, therefore, imperative to ensure that any exposures among animals are identified and treated, because without treatment, the likelihood of death is high. As harms related to illicit substance use continue to trend upward, recognizing the potentially fatal effects on animals in the surrounding environment may help to minimize exposure to household pets. Limitations of this study include presenting count data and not how exposures occurred and the data being limited to calls received, which is not generalizable to cats or dogs exposed to illicit stimulants overall.

## Notes

Supplement.
Data Sharing Statement
