# Supplementary material for: Cat and Dog Exposures to Cocaine or Methamphetamine
Source: JAMA Netw Open. 2024 Dec 20;7(12):e2451833. doi: 10.1001/jamanetworkopen.2024.51833 (PMC11662247; doi:10.1001/jamanetworkopen.2024.51833)
Supplement: Supplement. — Data Sharing Statement [file jamanetwopen-e2451833-s001.pdf]

Data Sharing Statement

Ware. Cat and Dog Exposures to Cocaine or Methamphetamine. *JAMA Netw Open*. Published December 20, 2024. doi:10.1001/jamanetworkopen.2024.51833

Data
Data available: No

Additional Information
Explanation for why data not available: The data are proprietary and owned by SafetyCall International, LLC and Pet Poison Helpline. Due to the proprietary and confidential nature of the data, they will not be made publicly available or shared.
